# Associations between I/D polymorphism in the *ACE* gene and lung cancer: an updated systematic review and a meta-analysis

**DOI:** 10.1186/s12885-021-07825-5

**Published:** 2021-02-12

**Authors:** Junjian Chen, Mao Sun, Min Zhou, Renfu Lu

**Affiliations:** Department of Cardiothoracic Surgery, Chongqing Emergency Medical Center, Chongqing University Center Hospital, No.1 Healthy Road, Yuzhong District, Chongqing, 400014 China

**Keywords:** *ACE*, Polymorphism, Lung cancer, Meta-analysis

## Abstract

**Background:**

We evaluated the association between the I/D polymorphism in the *ACE* gene and lung cancer risk by performing a meta-analysis.

**Methods:**

The heterogeneity in the study was tested using the Cochran χ^2^-based Q statistic test and I^2^ test, and then the random ratio or fixed effect was utilized to merge the odds ratios (ORs) and 95% confidence intervals (CIs) to estimate the strength of the association between *ACE* polymorphisms and susceptibility to lung cancer. Sensitivity analysis was also performed. Using funnel plot and Begg’s rank test, we investigated the publication bias. All statistical analyses were performed using Stata 12.0 and RevMan 5.3.

**Results:**

A total of 4307 participants (2181 patients; 2126 controls) were included in the 12 case–control studies. No significant association was found between the *ACE* I/D polymorphism and lung cancer risk (II vs. ID + DD: OR = 1.22, 95% CI = 0.89–1.68; II + ID vs. DD: OR = 1.21, 95% CI = 0.90–1.63; I vs. D: OR = 1.15, 95% CI = 0.95–1.39). In the subgroup analysis by ethnicity, no significant association between the *ACE* I/D polymorphism and lung cancer risk was found among Asian and Caucasian populations for the comparisons of II vs. ID + DD, II + ID vs. DD, and I vs. D genetic models.

**Conclusion:**

The *ACE* I/D polymorphism is not associated with the risk of lung cancer.

## Background

Lung cancer has become the largest malignant tumor in terms of harm to human health and life [1, 2]. The incidence and mortality of lung cancer are also increasing every year, and its proportion in tumor mortality has also expanded [2]. The 5-year survival rate of lung cancer is only approximately 15% [3].

The human angiotensin-converting enzyme (*ACE*) gene, located on chromosome 17q23, has a length of 21 kb and consists of 26 exons and 25 introns [4]. The biological function of the *ACE* gene is affected by an insertion/deletion in the 16th intron, producing three genotypes: insertion homozygous (II), deletion homozygous (DD), and insertion/deletion of heterozygous type (ID) [5]. *ACE* activity in serum is related to the I/D polymorphism of the *ACE* gene [6]. The II genotype exhibits low activity, the DD gene exhibits high activity, and the heterozygous ID genotype has activity between the two [7].

In recent years, there have been many studies on the role of *ACE* I/D polymorphism in the risk of lung cancer, but there were some contradictions among the results of these studies. Some studies showed an obvious trend of the *ACE* ‘II’ genotype with increased risk of lung cancer [8, 9], whereas other studies have shown that the DD genotype of *ACE* contributes to a higher risk of lung cancer [10–13]. However, another study has shown that the *ACE* ‘ID’ genotype might increase the risk of lung cancer [14]. In addition, other studies have shown no association between *ACE* I/D polymorphism and lung cancer [15–19]. To more accurately assess the potential relationship between the *ACE* I/D polymorphism and the risk of lung cancer, we performed a meta-analysis using all eligible published studies.

## Methods

### Search strategies

We conducted a comprehensive search of the literature in the Web of Science, PubMed, Cochrane Library, Embase, and China National Knowledge Infrastructure (CNKI) electronic databases, covering relevant studies published as of June 31, 2019. The keywords for the search were as follows: (“angiotensin-converting enzyme” OR “*ACE*”) AND (“polymorphism” OR “variant” OR “mutation”) AND (“Lung cancer” OR “lung neoplasm”’). The literature on relevant data was searched in English and Chinese. In addition, retrieved articles and references were manually searched. Referring to the Preferred Reporting Project (PRISMA) Guide for Systematic Evaluation and Meta-Analysis [20], an information flow diagram related to the final eligibility data was constructed by screening all retrieved studies.

### Inclusion and exclusion criteria

Screening for the studies of the relationship between *ACE* I/D polymorphism and the risk of lung cancer was performed according to the following inclusion criteria: (1) the design of the study was case–control; (2) the full text can be found; (3) the genotype information of the *ACE* I/D polymorphism was available; (4) the relationship of the *ACE* I/D polymorphism and the risk of lung cancer was evaluated. The major exclusion criteria were: (1) not a case–control study; (2) repeating early publications (studies used in different publications for the same sample data, including only the most complete samples after careful review); (3) unpublished articles, conference papers, meta-analysis, and systematic reviews; (4) family-based pedigree research. This meta-analysis strictly followed the requirements of PRISMA [20].

### Data extraction

The data of the selected studies were independently extracted by two researchers using standard data collection forms. The information extracted from the literature was as follows: first author, year of publication, country of origin, mean age and gender in cases and controls, number of cases and controls, Hardy-Weinberg equilibrium, genotyping method, source of controls, and available genotype frequency information for *ACE* I/D. If the same sample data appeared in multiple publications, only publications with the largest sample size were included in the study. The differences between the two investigators were resolved through discussion. If the discussion could not resolve the objection between the two researchers, the objection was judged by a third investigator. All data were obtained from the full text of the published research, and the authors were not contacted for further information. All information on the participants in the selected studies is presented in Table 1.

**Table 1:** Characteristic of studies included in the meta-analysis

Author	year	country	Ethnicity	Age group	Genotype Methods	Source of control	NOS score	HWE
Peddireddy et al [14]	2018	South Indian	Asia	Adult	PCR	PB	8	0.726
Phukan et al [9]	2014	Northeast India	Asia	Adult	PCR	PB	8	0.227
Ozen et al [16]	2013	Turkey	Caucasians	Adult	PCR	PB	7	0.920
Shi et al [12]	2014	China	Asia	Adult	PCR-SSP	PB	6	0.308
Cheon et al [15]	2000	Korea	Asia	Adult	PCR	–	6	0.133
Yaren et al [17]	2008	Turkey	Caucasians	Adult	PCR	–	7	0.470
Nacak et al [8]	2010	Turkey	Caucasians	Adult	PCR	PB	8	0.268
Wang et al [13, 21]	2000	China	Asia	Adult	PCR	–	6	0.861
Zhang et al [18]	2005	China	Asia	Adult	PCR	HB	7	0.109
Gao et al [11]	2012	China	Asia	Adult	PCR	HB	6	0.018
Devic Pavlic et al [10]	2012	Croatia	Caucasians	Adult	PCR	HB	7	0.909
Ding et al [19]	2008	China	Asia	Adult	PCR	HB	7	0.175

### Study quality assessment

Two evaluators evaluated the quality of the included studies according to the Newcastle-Ottawa Scale (NOS) [22], which is applicable to the quality assessment of observational studies. The difference between the two evaluators was reported and resolved by a third evaluator. The scores of research quality included mainly the following three aspects: (1) selection of the case groups and control groups (4 stars); (2) quality of confounding factor correction in the case and control populations (2 stars); and (3) determination of the exposure of interest in the studies (3 stars). For each item numbered in the selection and exposure categories, one study can be rated as up to one star, and comparability can be assigned up to two stars. Higher scores indicated an increase in the quality of the study. Studies with scores equal to or higher than six were considered high-quality studies.

### Data analysis

The heterogeneity in the study was tested using the Cochran χ^2^-based Q statistic test and I^2^ test, and then the random ratio or fixed effect was utilized to merge the odds ratios (ORs) and 95% confidence intervals (CIs). The significance of the pooled OR was analyzed by the Z-test (*P* < 0.05, judged statistically significant). To estimate the strength of the association between *ACE* polymorphisms and susceptibility to lung cancer, we performed a sensitivity analysis. Using funnel plot and Begg’s rank test, publication bias was investigated. All statistical analyses were performed using Stata 12.0 (Stata Corp, College Station, TX, USA) and RevMan 5.3.

## Results

### Literature search and study characteristics

**Fig. 1:**
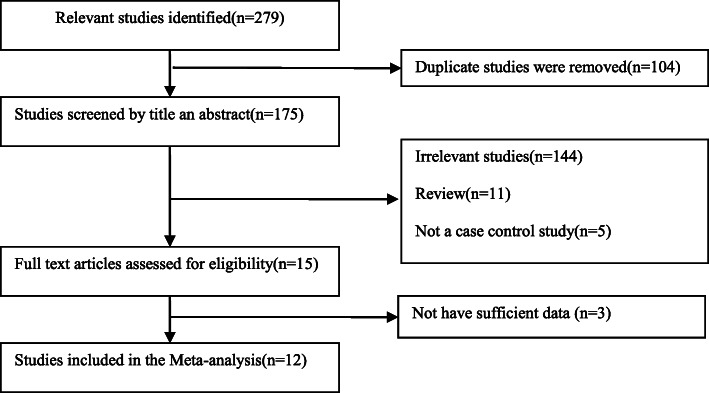
The flow sheet of identification of eligible studies

A flow chart of the literature search is shown in Fig. 1. A total of 279 potentially relevant articles were selected for the preliminary online search. After verifying and deleting 104 duplicate articles, 175 articles were included for further consideration. Through a review of the titles and abstracts, 15 articles were included for full-text review. Finally, 12 articles were included in the final analysis. These studies were published between 2005 and 2018, and included 2181 patients with lung cancer and 2126 controls. Except for one study, the distribution of genotypes in the controls followed the Hardy–Weinberg equilibrium (HWE). In addition, the NOS scores for all the studies ranged from 6 to 8 points; thus, the selected articles were considered to have high methodological quality. The relevant information of the included articles is shown in Tables 1 and 2.

**Table 2:** The genotype distribution of *ACE* I/ D

Author	Sample size (case/control)	Female (%) (case/control)	case					control				
			I/I	I/D	D/D	I	D	I/I	I/D	D/D	I	D
Peddireddy et al [14]	246/250	28.0/28.0	48	161	37	257	235	111	113	26	335	165
Phukan et al [9]	151/151	45.7 /45.7	61	62	28	184	118	44	68	39	156	146
Ozen et al [16]	52/212	11.5/−	10	30	12	50	54	67	105	40	239	185
Shi et al [12]	120/62	39.2/−	47	49	24	143	97	26	31	5	83	41
Cheon et al [15]	218/121	26.6/42.1	72	116	31	260	178	48	50	23	146	96
Yaren et al [17]	75/85	8.0/9.4	4	39	32	47	103	14	37	34	65	105
Nacak et al [8]	125/165	12.0/48.5	37	50	38	124	126	29	72	64	130	200
Wang et al [13, 21]	34/38	23.5/44.7	10	6	18	26	42	13	18	7	44	32
Zhang et al [18]	47/54	14.9 /29.6	21	21	5	63	31	20	30	4	70	38
Gao et al [11]	684/602	27.2/33.6	351	271	62	973	395	320	253	29	893	311
Devic Pavlic et al [10]	308/353	29.5/38.5	64	148	96	276	340	78	177	98	333	373
Ding et al [19]	121/33	31.4/30.3	55	56	10	166	76	19	10	4	48	18

### Meta-analysis results

**Fig. 2:**
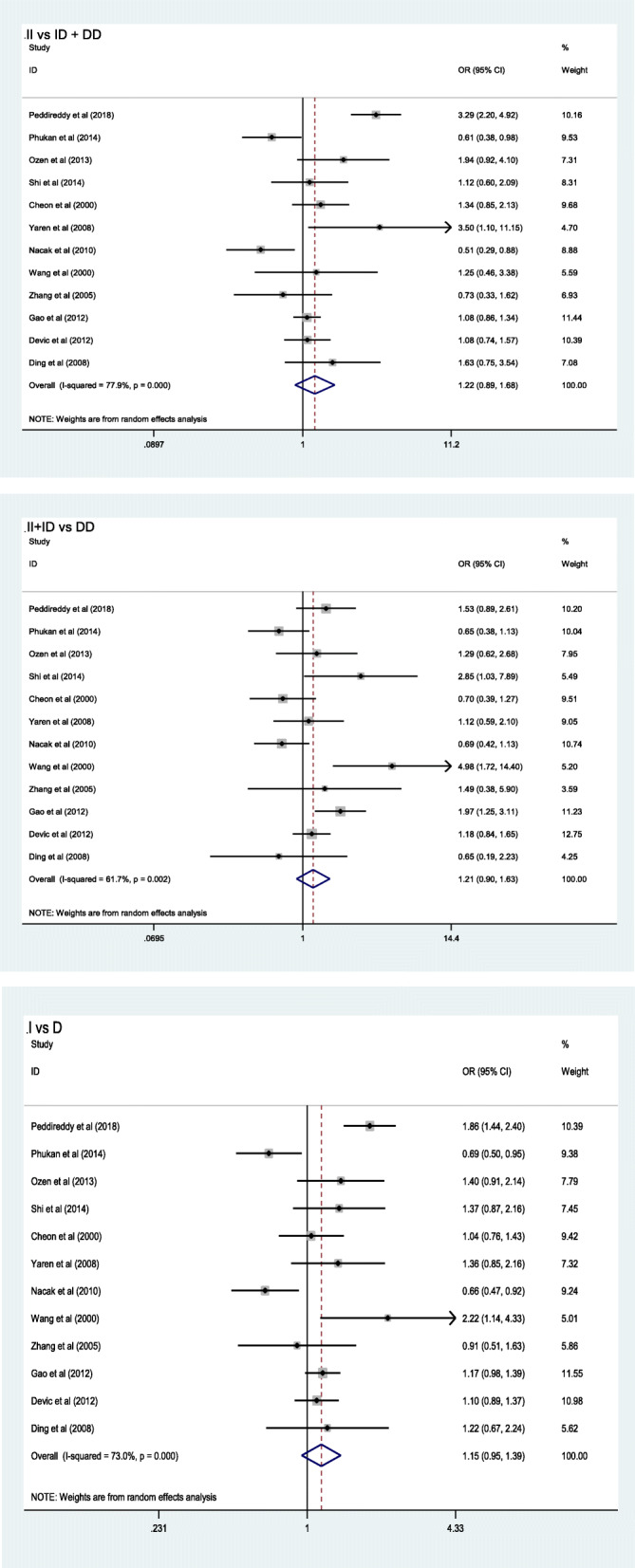
Forest plot results of meta- analysis for association between the *ACE* I/D polymorphism and lung cancer under different genetic models (II vs ID+DD; II + ID vs DD; I vs D)

**Fig. 3:**
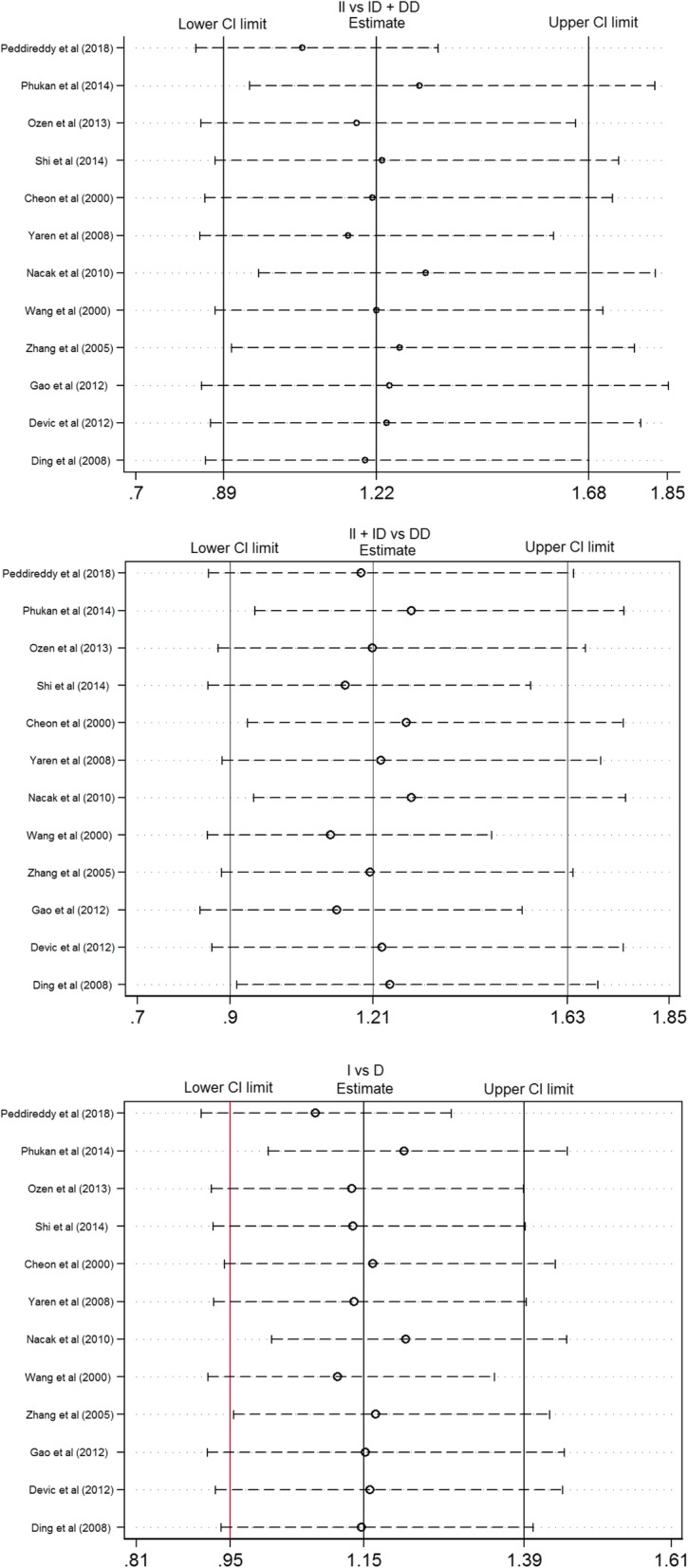
Sensitivity analysis examining the association between the *ACE* I/D polymorphism and risk of lung cancer under three model (II vs ID+DD, II + ID vs DD, I vs D)

**Fig. 4:**
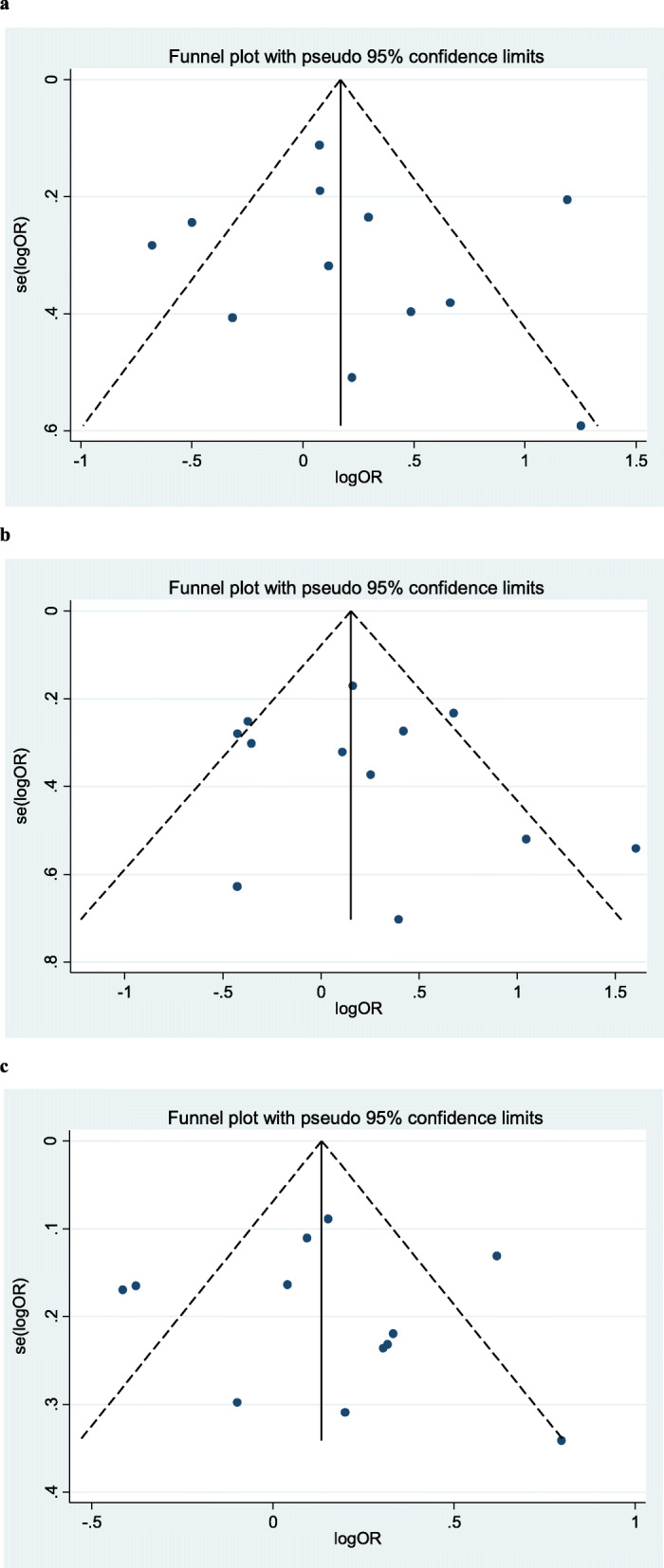
Begg’s funnel plot for publication bias analysis. a is the model of II vs ID+DD; b is the model of II + ID vs DD; c is the model of I vs D

The heterogeneity of the three genetic models was determined using the Q test and I squared statistics. As shown in Fig. 2, there was significant heterogeneity in the three models (II vs. ID + DD: *P* < 0.001, *I*^*2*^ = 77.9%; II + ID vs. DD: *P* = 0.002, *I*^*2*^ = 61.7%; I vs. D: *P* < 0.001, *I*^*2*^ = 73.0%); thus, the random-effect model was employed in the analysis of the three models. Our results revealed that there were no significant associations between the *ACE* I/D polymorphism and lung cancer in the II vs. ID + DD (OR = 1.22, 95% CI = 0.89–1.68, *P* = 0.22), II + ID vs. DD (OR = 1.21, 95% CI = 0.90–1.63, *P* = 0.21), and I vs. D (OR = 1.15, 95% CI = 0.95–1.39, *P* = 0.15). In the subgroup analysis by ethnicity, no significant association was found among the three models in both Caucasian and Asian populations (Table 3). Sensitivity analysis was used to assess the impact of each individual study on the pooled OR by sequentially removing each eligible study. Our results suggest that none of the studies affected the overall outcome of the pooled OR (Fig. 3). Begg’s funnel plot was used to assess publication bias, and the results showed that publication bias was not reflected in the three genetic models. (II vs. ID + DD: *P* = 0.41; II + ID vs. DD: *P* = 0.34; I vs. D: *P* = 0.89) (Fig. 4).

**Table 3:** Summary of pooled OR in different ethnicities

Genetic model	group	Pooled OR (95% CI)	Heterogeneity		Test for overall effect	
			P	I^2^	Z	P
II vs ID+DD	Caucasians	1.22 (0.75–2.00)	0.007	71.9%	0.80	0.423
	Asia	1.21 (0.76–1.94)	< 0.01	82.9%	0.81	0.419
II + ID vs DD	Caucasians	0.98 (0.79–1.23)	0.284	20.5%	0.15	0.881
	Asia	1.56 (0.94–2.59)	0.005	67.3%	1.71	0.088
I vs D	Caucasians	1.05 (0.82–1.34)	< 0.01	73.0%	0.39	0.694
	Asia	1.24 (0.92–1.66)	< 0.01	77.9%	1.41	0.159

## Discussion

Recent studies have reported that *ACE* may be involved in the development of tumors [23–25]. *ACE* is a key enzyme in the renin-angiotensin system, which converts angiotensin I to angiotensin II and inactivates bradykinin. The mechanism of action may be due to the fact that angiotensin II can stimulate the synthesis of DNA and protein in vascular smooth muscle cells and promote the synthesis and secretion of vascular endothelial growth factor [26]. In addition, it may be involved in tumor development through bradykinin that can increase the permeability of the cell membrane to electrolytes and peptides. ACE can inactivate bradykinin [27]; however, low levels of *ACE* in tumor tissues can promote the invasive growth of malignant tumors. An increasing number of studies have investigated the association between ACE I/D polymorphism and lung cancer risk; however, there are inconsistencies and conflicting results. To further assess the association between ACE I/D polymorphism and lung cancer risk, we performed a meta-analysis of 12 case–control studies, including 2181 cases and 2126 controls.

The results of this meta-analysis showed no significant association between the three genetic models and the development of lung cancer. Although previous studies have revealed that *ACE* may have a certain effect on the etiology of lung cancer, our results suggest that these effects may not be caused by *ACE* gene mutations. The exact pathogenetic role of ACE in the etiology of lung cancer remains unclear. Our results suggest that the ACE I/D polymorphism does not affect cancer risk. Moreover, considering that this polymorphism may affect serum *ACE* levels and ACE levels may affect the risk of lung cancer, the risk for lung cancer is not directly caused by *ACE* gene mutations. Therefore, future research is necessary to determine the association between *ACE* polymorphism, ACE levels, and cancer risk.

Previously, a meta-analysis of eight published studies [8, 10, 11, 13, 15, 17–19] performed by Cheng et al. [28], including 1612 cases and 1442 controls, showed that the *ACE* I/D polymorphism is not associated with lung cancer. Wang et al. [21] have also conducted a meta-analysis of six published studies with 807 cases and 816 controls [8, 10, 13, 15, 18], and the results also showed that the *ACE* I/D polymorphism may not be associated with lung cancer risk. In our meta-analysis, 12 studies were included with 2181 cases and 2126 controls. Therefore, the statistical power of the current analysis is better than those of the two previous meta-analyses. Compared with the other studies, this study is more comprehensive regarding the relationship between *ACE* I/D polymorphism and lung cancer risk. Despite the differences between the studies included in the analysis, the results of our study suggest that the *ACE* I/D polymorphism may not lead to cancer risk, which is consistent with the findings of Cheng and Wang.

However, there are certain limitations to our study. First, databases that include studies published only in Chinese and English language were selected for analysis, and studies in other languages or unpublished potential research are missing. Second, due to the lack of raw data, we were unable to assess the potential gene–gene and gene–environment interactions. Third, the meta-analysis included data from Europeans and Asians, so the results apply only to these two ethnic groups. Fourth, among the three models, heterogeneity may greatly influence the conclusions of the meta-analysis. Lastly, the power of our statistical analysis with the current sample size was not high enough, so the results obtained from this study should be verified in the future.

## Conclusion

In summary, our study showed that *ACE* I/D polymorphism did not increase or decrease the risk to lung cancer. Further well-designed studies should be conducted to confirm our findings in different populations and age groups, such as different races in Asia or Europe or other populations or children and adults. Future research will also need to explore the possible role of *ACE* I/D gene–gene and gene–environment interactions in the susceptibility to lung cancer.

## Data Availability

All data generated or analyzed during this study are included in this published article.

## Abbreviations

OR: Odds ratios
CI: Confidence interval
ACE: Angiotensin-converting enzyme
CNKI: China Nationa Knowledge Infrastructure
NOS: Newcastle-Ottawa Scale
HWE: Hardy Weinberg equilibrium
