# Correction to: Associations between I/D polymorphism in the *ACE* gene and lung cancer: an updated systematic review and a meta-analysis

**DOI:** 10.1186/s12885-021-07937-y

**Published:** 2021-02-25

**Authors:** Junjian Chen, Mao Sun, Min Zhou, Renfu Lu

**Affiliations:** Department of Cardiothoracic Surgery, Chongqing Emergency Medical Center, Chongqing University Center Hospital, School of Medicine, Chongqing University, No.1 Healthy Road, Yuzhong District, Chongqing, 400014 China

**Correction to: BMC Cancer 21, 158 (2021)**

**https://doi.org/10.1186/s12885-021-07825-5**

Following publication of the original article [1], the authors reported an error in their affiliation.

The corrected affiliation is: Department of Cardiothoracic Surgery, Chongqing Emergency Medical Center, Chongqing University Center Hospital, School of Medicine, Chongqing University, No.1 Healthy Road, Yuzhong District, Chongqing, 400014 China

The original article [1] has been corrected.
